# Redesigning mental health research systems from within: the role of peer-led co-production

**DOI:** 10.3389/frhs.2025.1712015

**Published:** 2025-12-02

**Authors:** Nicole Anne D’souza, Sandy Rao, Kirsten Marchand, Megan Davies, Nicole S. J. Dryburgh, Ashley D. Radomski, Pankhuri Aggarwal, Christine Mulligan, Jillian E. Stringer, Amelia Austin, Jordan Edwards, Michelle D. Leach

**Affiliations:** 1 University of Toronto Dalla Lana School of Public Health, Toronto, ON, Canada; 2 Jack.org, Toronto, ON, Canada; 3 University of Calgary, Calgary, AB, Canada; 4 The University of British Columbia, Vancouver, BC, Canada; 5 Foundry, Vancouver, BC, Canada; 6 McGill University, Montreal, QC, Canada; 7 McMaster University, Hamilton, ON, Canada; 8 Harvard University, Cambridge, MA, United States; 9 Offord Centre for Child Studies, Hamilton, ON, Canada; 10 Children’s Hospital of Eastern Ontario, Ottawa, ON, Canada; 11 Knowledge Institute on Child and Youth Mental Health and Addictions, Ottawa, ON, Canada; 12 University of Cincinnati, Cincinnati, OH, United States; 13 University of Guelph, Guelph, ON, Canada; 14 Mathison Centre for Mental Health Research and Education, Calgary, AB, Canada; 15 University of Calgary Hotchkiss Brain Institute, Calgary, AB, Canada; 16 Hamilton Health Sciences, Hamilton, ON, Canada; 17 Yukon University, Whitehorse, YT, Canada

**Keywords:** co-production, mental health research, early career researchers (ECRs), collaborative research, institutional culture, health systems transformation, equity and inclusion, relational leadership

## Abstract

Momentum is building across mental health research and practice toward collaborative, equity-driven approaches, yet institutional cultures often remain rooted in hierarchy and individual achievement. Co-production has emerged as a framework for redistributing power, fostering reciprocity, and embedding lived experience into research and system design. However, it is typically framed as something that occurs externally with communities, while the internal research dynamics within institutions go unexamined. In this commentary, we argue that peer-led co-production is a vital but under-recognized strategy for transforming mental health systems from within. Drawing on the experience of the Network for Early Career X Trainee Researchers in Youth Mental Health (NExT) in Canada, we examine how early career researchers (ECRs) are modelling alternative ways of working through relational leadership, shared accountability, and collaborative infrastructures. We identify structural barriers that constrain this work, including siloed training pathways, narrow professional evaluation metrics, rigid role definitions, and funding mechanisms that undervalue relational practices. Building on these insights, we outline a roadmap for embedding peer-led co-production within institutions, calling for four shifts: sustained investment in relational infrastructure; training that embeds collaborative competencies; evaluation systems that reward both outcomes and processes; and leadership models that support shared governance. Peer-led networks demonstrate that these shifts are not abstract ideals but viable practices already in motion. Realizing their potential requires institutional commitment to reconfiguring funding, training, evaluation, and leadership so that co-production becomes foundational of mental health research and system change.

## Introduction

There is growing momentum within mental health systems research towards more collaborative, person-centered, and equity-driven ways of working (1). Terms such as co-design, co-creation, and co-production have gained prominence as researchers, health professionals, and policymakers seek to build more responsive and adaptable systems grounded in lived experiences. Yet, these terms are often used interchangeably, despite differences in intent, timing, and degree of involvement (2–4). In this perspective piece we focus on co-production, defined as a collaborative process grounded in shared power and decision-making across all phases of a project (5). Co-production emphasizes reciprocity, mutual learning, shared accountability, and relationship-building, with researchers working in equitable partnership from priority-setting through to knowledge sharing (2).

Too often, however, co-production is framed as something that happens “out there” with communities, while the internal hierarchies of our own institutions go unquestioned. As Masterson and Laidlaw (6) describe, co-production is fundamentally relational, promoting reflection, other-orientedness, and intentionality. These values matter not only for how we engage communities but also for how we collaborate within research teams, academic departments, and the broader professional and institutional cultures we are part of. In this sense, co-production is as much about culture as it is about method. It calls for reimagining how knowledge is generated, how credit and leadership are shared, and how institutions can become more equitable from the inside out.

Here, we propose that peer-led co-production offers a vital yet under-recognized strategy for transforming mental health systems and research from within. Drawing on the experience of the Network for Early Career X Trainee Researchers in Youth Mental Health (NExT), a collective of early career researchers (ECRs) committed to advancing youth mental health through equity, inclusion, and shared leadership, we examine how peer-led networks can serve as infrastructures for embedding relational, equity-focused ways of working into the everyday cultures of mental health research and practice.

## Peer-led networks as infrastructure for system change

One way the principles of co-production are already being enacted within mental health research and practices is through peer-led networks. These are collectives organized and governed by peers, such as ECRs, practitioners, or lived-experience leaders, who come together to share leadership, support one another, and advance change outside of traditional hierarchies. Such networks are more than professional development spaces; they embed equitable, relational ways of working into institutional culture (7, 8). Yet hierarchical structures continue to dominate mental health research and practice, often privileging top-down decision-making, gatekeeping, and rigid roles that concentrate authority among senior researchers, clinicians, and administrators. As Rose and Kalathil (9) observe, co-production often unfolds within systems that maintain existing power relations, where professional and academic voices continue to determine what constitutes legitimate knowledge. Wilde (10) similarly argues that these epistemic hierarchies within public mental health sustain structural oppression by privileging biomedical and managerial logics while marginalizing experiential and relational forms of knowing. These dynamics are not confined to service delivery; they are embedded within academic research cultures that reward individual productivity and seniority, leaving limited space for the collective, equity-oriented work that co-production requires. Within such environments, ECRs, often already engaged in co-design and community-based work, yet within institutional contexts that rarely support or sustain these approaches (24).

Peer-led networks create “horizontal spaces” where ECRs collaborate across roles, develop shared agendas, and navigate institutional constraints together. Authority and power are distributed rather than concentrated, challenging the notion that expertise flows from senior to junior members. In these settings, ECRs can practice leadership and relational accountability, skills that are essential to co-production but rarely taught, rewarded, or resourced within traditional academic or clinical pathways. Similar insights have been noted in education, where Russell et al. (11) show that professional learning communities provide the relational and reflective infrastructure that enable broader improvement networks to succeed. In this way, peer-led networks serve as microcosms of the responsive, adaptive, and community-driven systems that the mental health sector increasingly aspires to build.

NExT illustrates this in practice. Monthly peer-led monthly meetings create space for relationship-building, mutual support, collective idea generation, and adaptive problem-solving. Members bring projects and opportunities to the group, fostering discussions about system change and how to advocate for it together. Participation is voluntary yet consistent, underscoring the value members place on shared leadership and mutual learning. Structured around participatory processes, NExT reimagines research communities as inclusive, values-driven, and collaborative, challenging competitive cultures that silo knowledge and often fail to translate findings into meaningful system change. By investing in peer-led networks, we can create the conditions for co-production to take root, not just at the level of individual projects, but within the everyday work cultures of mental health systems themselves.

## Structural barriers to embedding co-production

While co-production holds promise as a transformative approach to mental health systems, its integration into the everyday work of researchers, particularly ECRs, is constrained by deep-rooted structural and institutional barriers.

Training pathways in mental health research still reflect siloed disciplinary traditions. Clinical programs prioritize diagnostic and therapeutic expertise, while research programs emphasize methodological rigor and productivity metrics such as publications and grant funding (12). Even in programs that aim to balance these priorities, few provide intentional opportunities to develop the relational competencies and systems literacy needed for co-production, community engagement, or cross-sector collaboration. Even in interdisciplinary contexts, co-production is typically treated as an optional rather than foundational (13).

Institutional reward structures reinforce these limitations. Academic success remains measured by individual outputs rather than collaborative processes or community impact. Initiatives such as the San Francisco Declaration on Research Assessment (14) and the Leiden Manifesto (15), have called for broader criteria that recognize openness, quality, and societal relevance, implementation within mental health research and training systems has been slow. Emerging practices like the Canadian Institutes of Health Research (CIHR) narrative CV (16) aim to capture contributions such as mentorship, collaboration, and community engagement, yet traditional metrics continue to dominate.

Institutional cultures add further constraints. Risk-aversive institutions often favor established methods and topics seen as safer or more fundable, undervaluing the time and resources needed for meaningful community engagement and alternative dissemination. Short-term funding further constrains ECRs' ability to sustain the trust and continuity needed for lasting partnerships. As a result, scholars pursuing community-based research, particularly those from equity-deserving groups, often struggle for recognition in systems that implicitly devalue lived experience and alternative ways of knowing.

Leadership opportunities are also limited. Decision-making often resides with senior colleagues, while few advancement pathways reward the relational and cross-sector expertise co-production requires. In addition, rigid role definitions within academic and clinical institutions limit flexibility in how ECRs work and with whom they can form partnerships. These divisions are particularly acute for those in embedded or partnered academic positions, who may be expected to meet the same academic demands as tenure-stream faculty while holding full-time clinical or health system roles, yet without equal access to institutional benefits or recognition. Such inequities reinforce hierarchies and restrict opportunities for meaningful collaboration. As a result, ECRs are often excluded from the strategic spaces where co-production could be embedded into research planning, evaluation, or program design.

Many researchers also occupy hybrid or intersecting roles (e.g., such as clinician-researchers, practitioner-scholars, or researchers with lived experience) that position them uniquely to bridge the cultures of research, practice, and community. These hybrid positions can facilitate co-production by fostering empathy, translational insight, and relational accountability (17). Yet they can also expose tensions within hierarchical institutions where legitimacy and advancement are still measured by traditional academic and professional norms (9). Navigating these dual expectations often requires researchers to translate across institutional logics, a form of boundary-spanning work that remains undervalued in most research systems. Recognizing and supporting these hybrid roles is essential to embedding co-production meaningfully within both academic and health system structures.

Funding mechanisms further shape what kinds of research are possible, and for whom. Existing grants tend to prioritize short-term, project-based outputs, leaving little room for the iterative, trust-based, and relational work that co-production entails. ECRs are often ineligible to serve as nominated principal applicants, especially when working across institutions or in collaboration with non-academic partners. This limits their ability to formally hold funding, gain visibility, and develop leadership within their institutions.

Many grants also fail to support the essential infrastructure of peer-led collaboration. Activities like collective planning, reciprocal mentorship, shared decision-making, and compensation for community partners are rarely seen as fundable deliverables, even though they are foundational to effective co-production. When these elements are unfunded or undervalued, ECRs are often forced to pursue them “off the side of their desks”, alongside their primary research and teaching responsibilities, reinforcing the perception that co-production is peripheral rather than central. Addressing these barriers requires rethinking how research capacity is developed, evaluated, and funded. Without targeted reforms, ECRs will remain constrained in their ability to fully participate in, lead, and embed co-production.

## Discussion: embedding co-production values in how we work as researchers

If co-production is to be taken seriously as a system-wide approach, its values must be reflected not only in what we study or who we involve, but also in how we work together. As ECRs working in the youth mental health sector, we are familiar with the conditions that foster well-being: trust, psychological safety, reciprocity, and inclusion. These values underpin key approaches across the sector, including trauma-informed care, equity-based models, and culturally grounded practice (6, 18). Yet when we step into our institutional roles, these same values are often left behind. We may advocate for rest and psychological safety for young people and communities while tolerating burnout, precarity, and competition in our own teams (19). We may call for power-sharing with service users while reinforcing power hoarding among senior researchers. Co-production cannot flourish if organizational cultures reproduce the very hierarchies and harms we seek to change.

Peer-led networks offer a powerful alternative. Grounded in principles of shared leadership and distributed accountability, they model the cultural shifts needed for co-production to take root in our institutions (6, 20). Kjellström et al. (20) identify nine relational practices that underpin effective co-production: intentional initiation, implementation of outcomes, power sharing, training and development, provision of support, investment in trusting relationships, open dialogue, networking, and coordination. These practices are central to how we organize, collaborate, and care for one another.

The NExT network illustrates what this looks like in practice. Leadership rotates according to members' interests, capacity, and professional development goals. One member might lead a conference presentation, while another coordinates a publication on the same topic. This distributes responsibility, supports skill development, protects against burnout, and increases efficiency by avoiding bottlenecks that arise in more hierarchical structures.

Intentional initiation in NExT begins with project ideas introduced in monthly meetings as open invitations, signaling voluntary, flexible, and respectful participation. Training occurs through co-authoring, co-facilitating, and mentoring, while emotional and logistical support is built into routine check-ins. Members support each other through career transitions, research dissemination, and navigating institutional systems.

Trusting relationships are built slowly and intentionally over time. Peers protect these relationships by allowing each other to opt in and out of projects without penalty. Stepping back due to burnout or competing demands does not limit future involvement, a principle that prioritizes people over productivity. Open dialogue allows disagreement and reflections are welcomed as part of the process, not as detours. This culture of openness makes it possible to address power imbalances or unmet needs directly. Coordination and networking are woven into everyday interactions, as members share resources, amplify each other's work, and connect across sectors and geographies. This creates a dynamic infrastructure for long-term collaboration beyond any one project. Implementation of outcomes is not only about deliverables but also by strengthened partnerships, new leadership capacities, and sustained cultural shifts.

By practicing these nine relational elements, peer-led networks demonstrate that co-production is not only a methodology but also a way of relating: about noticing power, organizing care, and cultivating shared responsibility. Trust-building and communication form the connective tissue of long-term collaboration across roles, institutions, and time. If mental health systems are to become responsive, inclusive, and just, we must carry these values into every aspect of how we work together.

## Looking ahead: a roadmap for embedding peer-led co-production within our institutions

To transition peer-led co-production into an integrated approach, institutions must align their structures, policies, and cultures accordingly. Initiatives like NExT demonstrate that this is possible, showing ECRs enacting co-production values through collaborative, equity-oriented practices. Realizing co-production's systemic potential requires sustained institutional commitment. Four structural shifts are especially critical.

First, institutions and funders must invest in the internal infrastructure that sustains co-production, not only in partnerships with external interest holders but also within research teams and academic environments. Collective planning, open dialogue, and shared decision-making are foundational to co-production but remain poorly resourced. While targeted streams in Canada, such as CIHR's Planning & Dissemination grants or the Social Science and Humanities Research Council (SSHRC) Partnership programs, offer some support, these mechanisms are typically short-term or project-specific. Sustained investment in the everyday relational practices is needed to make co-production a systemic norm. Allowing ECRs to serve as principal applicants or co-leads on cross-sector projects is one concrete way to build this capacity.

Second, training and career pathways must reflect the competencies co-production requires. Most programs still prioritize disciplinary knowledge and technical skills while relational and systems-level competencies, such as facilitative leadership, community engagement, and interdisciplinary collaboration, are treated as optional. To build real capacity for co-production, these competencies must be integrated into core curricula and professional development. Career pathways should also create space for experiential learning in collaborative and community-based contexts, preparing ECRs to navigate the relational and cross-sector work that co-production entails. Without these adjustments, training environments will continue to produce researchers who are technically skilled but underprepared for the collaborative, equity-oriented approaches increasingly expected in mental health research and practice.

Third, evaluation and accountability systems must be reoriented to reflect co-production values. Current metrics reward short-term individual productivity (e.g., publications and grant counts) over collaborative, process-based contributions that occur over longer time frames. Institutions should broaden assessment criteria to include team-based outputs, engagement with communities, quality of partnerships, community or health system impact, and equity outcomes. This requires revising authorship norms, acknowledging co-leadership, and incorporating participatory indicators into performance reviews. Initiatives such as the Declaration on Research Assessment (DORA), the Leiden Manifesto, and the adoption of narrative CVs point in this direction, but their adoption across mental health research and training environments remains slow. In developing this paper, for example, we piloted a collaborative authorship self-assessment process (see Supplementary File S1), adapted from the CalcuAuthor system (21), the Contributor Roles Taxonomy (CRediT) (22), and principles from the Civic Laboratory for Environmental Action Research's guidelines on equitable author order (CLEAR) (23) to guide authorship order based on contributions and to reflect co-production values rather than hierarchy or convention. Until evaluation systems fully recognize both research outcomes and the processes through which they are achieved, co-production will remain undervalued.

Finally, leadership models must evolve to support shared and distributed governance. In many academic and clinical institutions, leadership remains tied to hierarchy, seniority, and positional authority. Co-production, however, depends on models where responsibility, accountability, and decision-making are shared across roles, disciplines, and career stages. Institutions must recognize horizontal leadership as a legitimate and effective mode of organizing research, and create structures that support it, whether through participatory governance models, co-leadership roles, or policies that value relational leadership. Cultural change will be required to normalize these approaches, but doing so is essential to embedding co-production into the everyday functioning of research and system operations.

## Conclusion

Transforming mental health research systems requires internal alignment with the principles of co-production. Equity, reciprocity, and shared responsibility must guide not only how researchers engage with communities *but also* how institutions structure relationships, distribute authority, and define success. Embedding peer-led co-production demands four structural shifts: (1) sustained investment in relational infrastructure, (2) training that equips researchers with collaborative competencies, (3) evaluation systems that reward both outcomes and processes, and (4) leadership models that enable shared governance. Peer-led networks such as NExT show that these practices are not abstract ideals but viable alternatives already in motion. With intentional design and institutional commitment, these approaches can disrupt entrenched hierarchies, strengthen connections between evidence and practice, and create inclusive and responsive systems.

## Data Availability

The original contributions presented in the study are included in the article/Supplementary Material, further inquiries can be directed to the corresponding author.
