# Supplementary material for: Redesigning mental health research systems from within: the role of peer-led co-production
Source: Front Health Serv. 2025 Dec 2;5:1712015. doi: 10.3389/frhs.2025.1712015 (PMC12705551; doi:10.3389/frhs.2025.1712015)
Supplement: Supplementary file 1 [file Datasheet1.pdf]

NExT Authorship Self-Assessment Form

This form has been developed to support transparent, equitable, and values-aligned authorship practices within projects initiated through the NExT network. Drawing on the CalcuAuthor system (Martins et al., 2023), the Contributor Roles Taxonomy (CRediT) (NISO, n.d.), and principles articulated by the Civic Laboratory for Environmental Action Research (CLEAR), the tool provides a structured way to determine authorship order based on the nature and extent of individual contributions rather than hierarchy or convention.

Conventional authorship norms in academia frequently privilege seniority, institutional status, and disciplinary hierarchy, often obscuring significant intellectual, relational, and administrative labour. Such practices can disproportionately disadvantage trainees, early career researchers, community collaborators, and those undertaking equity-related or emotional labour. CLEAR highlights the importance of challenging these inherited hierarchies and recognizes that contributions such as community liaison work, facilitation, accessibility design, trust-building, and equity guardianship are often essential to collaborative research yet undervalued in traditional authorship models.

In alignment with co-production scholarship and NExT's guiding values of reciprocity, transparency, and shared accountability, this form explicitly acknowledges a broad spectrum of contributions. Authors are encouraged to reflect holistically on their involvement across the project lifecycle, including conceptualization, project coordination, relationship-building, data collection and analysis, writing, and critical review. The self-assessment scores serve as a foundation for an open team discussion about authorship order. Authorship decisions will be revisited at key milestones to reflect evolving roles, and any ties in contribution scores will be resolved by the Project Lead through collective dialogue.

Note: your answers in this section will be made public to the rest of the project team.

**Project Activities**	**Contribution Self-Assessment**<br>*Place an X in the column you feel best represents your contribution for each activity.*			
	**Major**:<br>I led or substantially contributed to this activity<br><br>1pt	**Moderate**:<br>I participated actively but did not lead<br><br>0.67	**Minor:**<br>I provided some input but had a limited role<br><br>0.33	**None**:<br>I did not contribute to this area.<br><br>0
**Research & Analytics**<br>Did I contribute to the collection, curation, analysis or synthesis of data in this project?				

**Project Administration** Did I contribute to the management and coordination of this project?				
**Conceptualization** Did I contribute to shaping the framework of this paper? (e.g., attending meetings, engaging in discussion, providing feedback on the ideas)				
**Writing - Introduction** Did I take responsibility for writing or developing content for this section?				
**Editing- Introduction** Did I take responsibility for editing or reviewing content for this section?				
**Writing- Methodology** Did I take responsibility for writing or developing content for this section?				
**Editing- Methodology** Did I take responsibility for editing or reviewing content for this section?				
**Writing - Results** Did I take responsibility for writing or developing content for this section?				
**Editing- Results** Did I take responsibility for editing or reviewing content for this section?				
**Writing - Discussion** Did I take responsibility for writing or developing content for this section?				

**Editing- Discussion** Did I take responsibility for editing or reviewing content for this section?				
**Writing - Conclusion** Did I take responsibility for writing or developing content for this section?				
**Editing- Conclusion** Did I take responsibility for editing or reviewing content for this section?				
**Data Visualization** Did I contribute to the preparation, creation, and presentation of data in visual formats (e.g., tables or figures)?				
**Critical Review** Did I conduct a critical review of one or more versions of the manuscript?				
**SCORE**				

Additional Questions:

These questions will not be used to calculate a self-assessment score but will help us develop an authorship statement for publication purposes, as well as better understand your contributions if there are decisions that need to be made related to authorship order.

Please select the authorship areas you feel you contributed to:

**Authorship Contribution Areas**	**Type an X in the column next to the activities you contributed to:**
Conceptualization	
Methodology	
Data Collection	

Data Analysis	
Writing - Original Draft	
Writing - Review and editing	
Funding Acquisition	

Please describe your contributions to this project in your own words (including anything you feel wasn’t captured already in this form):

**Short Answer:**
